# Supplementary material for: Factors associated with participation over time in the Avon Longitudinal Study of Parents and Children: a study using linked education and primary care data
Source: Int J Epidemiol. 2020 Oct 15;50(1):293–302. doi: 10.1093/ije/dyaa192 (PMC7938505; doi:10.1093/ije/dyaa192)
Supplement: dyaa192_Supplementary_Data [file dyaa192_supplementary_data.docx]

**Supplementary material**

[Factors associated with participation over time in the Avon Longitudinal Study of Parents and Children: a study using linked education and primary care data]

1. Questionnaires and clinics included in the analysis

Table S1: Timings of questionnaires and clinics included in our analysis

| Age (of child) | Mother-completed | Child-completed |
| --- | --- | --- |
| 8-42 weeks gestation | M |  |
| 12 weeks gestation | M |  |
| 18 weeks gestation | M |  |
| 32 weeks gestation | M |  |
| 4 weeks | Ch |  |
| 8 weeks | M |  |
| 6 months | Ch |  |
| 8 months | M |  |
| 15 months | Ch |  |
| 18 months | Ch |  |
| 21 months | M |  |
| 24 months | Ch |  |
| 30 months | Ch |  |
| 33 months | M |  |
| 38 months | Ch |  |
| 42 months | Ch |  |
| 47 months | M |  |
| 54 months | Ch |  |
| 57 months | Ch |  |
| 61 months | M |  |
| 65 months | Ch | Q |
| 69 months | Ch | Q |
| 73 months | M | Q |
| 77 months | Ch | Q |
| 81 months | Ch | Q |
| 85 months | M | Q |
| 7.5 years |  | Cl |
| 91 months | Ch | Q |
| 97 months | M | Q |
| 8.5 years |  | Cl |
| 103^1^ months | Ch | Q |
| 110 months | M | Q |
| 115 months | Ch | Q |
| 9.5 years |  | Cl |
| 122 months | M | Q |
| 10.5 years |  | Cl |
| 128 months | Ch | Q |
| 134 months | M | Q |
| 11.5 years |  | Cl |
| 140 months | Ch | Q |
| 145 months | M | Q |
| 12.5 years |  | Cl |
| 157 months | Ch | Q |
| 13.5 years |  | Cl |
| 166 months | Ch | Q |
| 167 months |  | Q |
| 169 months |  | Q |
| 15.5 years |  | Cl |
| 16.5 years | Ch | Q |
| 17.5 years |  | Cl |
| 18 years | M | Q |
| 19.5 years | Ch |  |
| 20 years |  | Q |

1. Two questionnaires about different topics were administered at the same time
2. Linkage to GP records

As part of the Secure Anonymised Information Linkage (SAIL) project [1], the NHS Wales Information Service (NWIS) and the Health Informatics Research Unit (HIRU) at the University of Swansea have established a method through which individual level data from multiple sources can be linked and analysed in a secure setting, including data from primary care electronic patient records. The Project to Enhance ALSPAC through Record Linkage (PEARL), in collaboration with the SAIL team and working with GP software system providers, developed two methods to extract GP records (where permissions allowed):

1. Pilot extraction: In 2012 ALSPAC carried out a pilot extraction of GP records of an initial pilot sample of index participants. The extraction took place from assenting GP practices across England & Wales. The methods for this extraction have been described in a previous paper [2].
2. Main extraction: The NHS South West Commissioning Support Unit (SWCSU) has developed a governance framework and data extraction mechanism which secured opt-in assent from GP practices for the extraction of records and their use for SWCSU approved purposes. Invitations to participate in this system were made to all practices in the Bristol, North Somerset, Somerset and South Gloucestershire (BNSSSG) clinical commissioning group. The extraction mechanism is provided by EMIS, which supplies software systems to the majority of practices in the BNSSSG area. ALSPAC gained approval from the SWCSU Security and Informatics Group to extract participants’ GP records. SWCSU informed all participating practices about this agreement and gave them opportunity to opt-out.

For both the pilot study and the main extraction, the methods after extraction were identical. The extracted records were pseudonymised at source and securely transferred into a copy of the SAIL secure setting (known as a UK Secure eResearch Platform, or UKSeRP) using SAIL’s “split file” method and adhered to NHS standards of encryption and security, as described previously [2].

1. Variables derived from GP data

BMI: Read codes (the UK GP coding system) 22K.. (BMI), 229.. (O/E - height), and 22A.. (O/E - weight) were used to define BMI. The mean of all measurements after age 10 was used; where there was only one measurement, this was used.

Consultation rate age 15-19 years: As others have done [3], we defined consultations by excluding any Read codes relating to administration, hospitalisations and provision of services and by counting multiple GP interactions within 1 day as one consultation.

(Prescribed) drug count age 15-19 years: As in previous research [4, 5], we counted the number of different drugs received by each participant at each year of age to provide an overall measure of morbidity. Each unique drug name was counted only once – so that repeat prescriptions and different formulations or doses of the same drug were not counted.

Smoking before age 18 years: Two recent studies have defined smoking status using Read codes [6, 7]. In the current study, we used a combined set of codes but omitted the codes 6791. (health education – smoking) as this appeared to result in a large number of false positives (for example, there were many occasions on which this was recorded alongside a code for never smoked). Similarly, if codes about smoking cessation advice (67H1., 67H6., 8CAL., and others) were recorded but the individual was concurrently recorded as having never smoked, then this instance was classified as not smoking. As in the study by Atkinson and colleagues [6], if a Read code required a value to be recorded (for example, number of cigarettes per day) then we only classified someone as a smoker according to this code if this value was non-missing and greater than zero. Using this definition, individuals were recorded – at one or more time points – as either a non-smoker, an ex-smoker or a smoker. From this we generated smoking status at age 18 years (ever smoked or never smoked).

1. Summary of missing data

Table S2: Number (%) with missing data for each variable (n=13 972)

| Variable | Number (%) missing |
| --- | --- |
| **Baseline variables** |  |
| Sex | 0 |
| Mother’s age at index birth | 0 |
| Age at first pregnancy | 829 (6%) |
| Smoked in pregnancy | 819 (6%) |
| Smoked ever | 922 (7%) |
| Marital status | 890 (6%) |
| Mother’s ethnicity | 1648 (12%) |
| Mother’s education | 1560 (11%) |
| Duration of breastfeeding | 1410 (10%) |
| Maternal antenatal depression score | 1998 (14%) |
| Phone in home | 901 (6%) |
| Car | 941 (7%) |
| Housing tenure | 950 (7%) |
| Number of rooms | 1083 (8%) |
| Crowding index | 1173 (8%) |
| Double glazing | 1504 (11%) |
| Financial difficulties score | 1889 (14%) |
| Family occupational social class | 2472 (18%) |
| **Education variables** |  |
| Key stage 4 attainment score | 2558 (18%) |
| School absence in year 11 | 3151 (23%) |
| SEN status in year 11 | 3162 (23%) |
| **Measures from GP data** | Number (%) missing of 13 972 [Number (%) missing among the n=10 811 with GP data beyond age 4 years] |
| Asthma before age 8 |  |
| Smoking before age 18 | 4988 (36%) [1827 (17%)] |
| Depression before age 18 | 5207 (37%) [2046 (19%)] |
| Mean BMI | 7040 (50%) [3879 (36%)] |
| Consultation rate 15-19 years | 5052 (36%) [1891 (17%)] |
| Prescription rate 15-19 years | 5052 (36%) [1891 (17%)] |

As stated in the paper, 9049 individuals (65% of the 13 972) had complete covariate data. As expected, these individuals had higher rates of participation compared to those with incomplete data (results in main text). There were 6984 individuals (50% of the original sample) with baseline covariates and linked education data. Those with linked education data in addition to baseline covariates did not differ from all those with complete baseline covariates with respect to participation (mean number of questionnaires completed/clinics attended: 32 for mothers, 19 for children among those with education data as well as baseline covariates). Finally, there were between 4280 and 6671 individuals with baseline covariates and linked GP data for the child and mother participation analysis. The mean number of questionnaires completed by mothers in this group was 34; the mean (questionnaires completed and clinics attended) for children was 21.

1. Multiple imputation models

Table S2 shows which variables were included in the two imputation models and in what form. Note that two separate imputation models were needed because the linked ALSPAC – GP data is stored on a secure server at Swansea University and excludes any individuals who have explicitly dissented to linkage to their health data (i.e. not all 13 972 study participants are included in the linked ALSPAC-GP dataset). Further the imputation model for the GP data only included individuals with GP data at least beyond the age of 4 (so that at least one of the GP variables – consultation and prescription rates aged 0-4 – was non-missing). In the first imputation model (model 1), IQ was imputed from the cube of the attainment score and the attainment score from the cube root of IQ. These variables were included as linear terms when imputing other variables. This has been described previously [8]. A square root transformation was applied to percent absence prior to imputation and it was used in its transformed form throughout the analysis.

Table S3: Variables included in the imputation models (model 1: n=13 972; model 2: n=10 811)

| Variable | Type of variable | Regression model / method used to impute this variable | Included in imputation model 1^1^? | Included in imputation model 2^2^? |
| --- | --- | --- | --- | --- |
| Sex | Binary | N/A | ✓ | ✓ |
| Mother’s age at index birth | Numerical | N/A | ✓ | ✓ |
| Age at first pregnancy | Categorical | Multinomial logistic | ✓ | ✓ |
| Smoked in pregnancy | Binary | Logistic | ✓ | ✓ |
| Smoked ever | Binary | Logistic | ✓ | ✓ |
| Marital status | Binary | Logistic | ✓ | ✓ |
| Mother’s ethnicity | Binary | Logistic | ✓ | ✓ |
| Mother’s education | Categorical | Multinomial logistic | ✓ | ✓ |
| Duration of breastfeeding | Categorical | Multinomial logistic | ✓ | ✓ |
| Antenatal depression score | Numerical | Predictive mean matching (PMM) | ✓ | ✓ |
| Phone in home | Binary | Logistic | ✓ | ✓ |
| Car | Binary | Logistic | ✓ | ✓ |
| Housing tenure | Categorical | Multinomial logistic | ✓ | ✓ |
| Number of rooms | Numerical | PMM | ✓ | ✓ |
| Crowding index | Categorical | Multinomial logistic | ✓ | ✓ |
| Double glazing | Binary | Logistic | ✓ | ✓ |
| Financial difficulties score | Numerical | PMM | ✓ | ✓ |
| Occupational social class | Binary | Logistic | ✓ | ✓ |
| Key stage 4 attainment score^3^ | Numerical | Linear regression | ✓ | ✓ |
| School absence in year 11^4^ | Numerical | Linear regression | ✓ | X |
| SEN status in year 11 | Categorical | Multinomial logistic | ✓ | X |
| Asthma diagnosis before age 8 | Binary | Logistic | X | ✓ |
| Smoking before age 18 | Binary | Logistic | X | ✓ |
| Depression before age 18 | Binary | Logistic | X | ✓ |
| Mean BMI | Numerical | Linear regression | X | ✓ |
| Consultation rate 15-19 years^5^ | Numerical | Linear regression | X | ✓ |
| Prescription rate 15-19 years^5^ | Numerical | Linear regression | X | ✓ |
| Attended age 3.5 examination | Binary | Logistic | X | ✓ |
| **Auxiliary variables** |  |  |  |  |
| Child IQ at 8 years^6^ | Numerical | Linear regression | ✓ | ✓ |
| Consultation rate 0-4 years | Numerical | N/A | X | ✓ |
| Consultation rate 5-9 years | Numerical | Linear regression | X | ✓ |
| Consultation rate 10-14 years | Numerical | Linear regression | X | ✓ |
| Consultation rate 20+ years | Numerical | Linear regression | X | ✓ |
| Prescription rate 0-4 years | Numerical | N/A | X | ✓ |
| Prescription rate 5-9 years | Numerical | Linear regression | X | ✓ |
| Prescription rate 10-14 years | Numerical | Linear regression | X | ✓ |
| Prescription rate 20+ years | Numerical | Linear regression | X | ✓ |

1. Model 1 was used to impute baseline and school variables and the resulting imputed data used to analyse the association between the baseline and education variables and participation.
2. Model 2 was used to impute baseline and GP variables and the resulting imputed data used to analyse the association between GP variables and participation (adjusting for baseline covariates). This model included only individuals with at least some GP data from age 5 years.
3. Attainment cubed included when imputing IQ; as linear term otherwise.
4. Transformed: square root of absence used throughout.
5. Categorical variable used in the analysis: passively imputed from the continuous version.
6. Cube root of IQ included when imputing attainment; as linear term otherwise.
7. Results from the complete case analysis

Table S4: Odds ratios for participation for all baseline covariates among complete cases (n=9,049)

|  |  | Child participation | p-value | Mother participation | p-value |
| --- | --- | --- | --- | --- | --- |
| Covariate | Level | OR (95% CI) |  | OR (95% CI) |  |
| Sex  Mother’s education  Parity  Mother’s age (at birth of index child)  Mother’s ethnicity  Family social class  Age at first pregnancy  Maternal smoking  Duration of breastfeeding  Married  Housing tenure  Number of rooms  Phone in home  Car use  Double glazing  Financial difficulties  Crowding index  Depression score | Female vs male  O level / lower  A level  Degree/higher  0  1  2+  Per 1 year increase  Non-white vs white  Manual vs non-manual  <20  20-24  25+  Yes vs no (in pregnancy)  Yes vs no (ever)  Never/<1 month  1 to <3 months  3 to <6 months  6 months+  Yes vs no  Owned/mortgaged  Private rented  Council/HA/other  Per 1 room increase  Yes vs no/incoming only  No vs yes  None vs full/partial  Per 1 unit increase  ≤0.5  >0.5 – 0.75  >0.75 – 1  >1  Per 1 unit increase | 1.87 (1.69, 2.08)  1.00  1.48 (1.30, 1.69)  1.76 (1.47, 2.09)  1.00  0.78 (0.67, 0.90)  0.58 (0.46, 0.72)  1.08 (1.06, 1.10)  0.53 (0.36, 0.78)  0.83 (0.72, 0.97)  1.00  1.39 (1.17, 1.65)  1.46 (1.21, 1.77)  0.81 (0.69, 0.95)  0.78 (0.69, 0.88)  1.00  1.72 (1.45, 2.04)  1.81 (1.55, 2.13)  2.24 (1.95, 2.57)  1.09 (0.94, 1.26)  1.00  0.62 (0.48, 0.79)  0.85 (0.70, 1.03)  1.03 (0.98, 1.09)  0.69 (0.55, 0.87)  0.69 (0.54, 0.87)  0.88 (0.79, 0.98)  0.98 (0.96, 1.00)  1.00  0.92 (0.79, 1.07)  0.82 (0.66, 1.02)  0.67 (0.49, 0.92)  0.99 (0.98, 1.00) | <0.001  <0.001  <0.001  <0.001  0.001  0.02  <0.001  0.008  <0.001  <0.001  0.3  <0.001  0.3  0.001  0.002  0.02  0.02  0.09  0.06 | 1.07 (0.95, 1.21)  1.00  1.66 (1.42, 1.94)  2.00 (1.63, 2.45)  1.00  0.76 (0.64, 0.90)  0.57 (0.44, 0.73)  1.09 (1.07, 1.11)  0.29 (0.18, 0.44)  0.72 (0.61, 0.85)  1.00  1.40 (1.15, 1.70)  1.72 (1.39, 2.14)  0.78 (0.65, 0.93)  0.82 (0.71, 0.95)  1.00  1.68 (1.38, 2.03)  1.74 (1.45, 2.09)  2.38 (2.03, 2.79)  1.11 (0.93, 1.31)  1.00  0.57 (0.43, 0.75)  0.81 (0.65, 1.01)  1.01 (0.95, 1.08)  0.69 (0.54, 0.90)  0.76 (0.59, 1.00)  0.86 (0.76, 0.98)  0.98 (0.96, 1.00)  1.00  0.83 (0.69, 0.99)  0.76 (0.59, 0.97)  0.57 (0.40, 0.82)  0.98 (0.97, 0.99) | 0.2  <0.001  <0.001  <0.001  <0.001  <0.001  <0.001  0.007  <0.001  <0.001  0.2  <0.001  0.7  0.005  0.05  0.02  0.02  0.06  0.003 |

Table S5: Odds ratios for participation among complete cases: education variables (n=6,984)

|  |  | Child participation | p-value | Mother participation | p-value |
| --- | --- | --- | --- | --- | --- |
| Covariate | Level | OR (95% CI)^1^ |  | OR (95% CI)^1^ |  |
| Attainment score  SEN status  School absence | for 10 point increase  None  School action  Statement  For 1 point increase in square root of % absence | 1.06 (1.05, 1.07)  1.00  0.80 (0.67, 0.96)  0.56 (0.38, 0.83)  0.88 (0.84, 0.92) | <0.001  0.003  <0.001 | 1.05 (1.04, 1.06)  1.00  0.94 (0.76, 1.17)  0.85 (0.54, 1.35)  0.87 (0.82, 0.91) | <0.001  0.7  <0.001 |

1. Mutually adjusted and adjusted for baseline factors.

Table S6: Odds ratios for participation among complete cases: GP-derived measures

|  |  | Child participation | p-value | Mother participation | p-value |
| --- | --- | --- | --- | --- | --- |
| Covariate |  | OR (95% CI)^1^ |  | OR (95% CI)^1^ |  |
| Asthma diagnosis by age 8^2a^  Smoking record by age 18^2b^  Depression before age 18^2c^  BMI^2d^  Consultation rate age 15-19^2e^  Prescription rate age 15-19^2e^ | Yes vs no  Yes vs no  Yes vs no  per 1kg/m  ≤1 per year  >1 – 4 per year  >4 per year  ≤1 per year  >1 – 4 per year  >4 per year | 6.05 (4.39, 8.32)  0.63 (0.53, 0.76)  0.71 (0.56, 0.90)  0.98 (0.96, 0.99)  1.00  1.48 (1.26, 1.75)  1.74 (1.45, 2.09)  1.00  1.41 (1.23, 1.62)  1.52 (1.25, 1.86) | <0.001  <0.001  0.005  0.001  <0.001  <0.001 | 5.91 (3.99, 8.75)  0.68 (0.55, 0.84)  0.80 (0.60, 1.07)  0.97 (0.95, 0.99)  1.00  1.23 (1.01, 1.50)  1.25 (1.00, 1.56)  1.00  1.19 (1.01, 1.40)  1.19 (0.94, 1.52) | <0.001  <0.001  0.1  0.001  0.09  0.1 |

1. Adjusted for baseline factors.
2. a) n=6671 & 6652; b) n= 5527 & 5513; c) n=5413 & 5399; d) n=4290 & 4280; e) n=5477 & 5464 for child and mother participation, respectively.

References

1. Ford, D.V., et al., *The SAIL Databank: building a national architecture for e-health research and evaluation.* BMC Health Serv Res, 2009. **9**: p. 157.

2. Cornish, R.P., et al., *Defining adolescent common mental disorders using electronic primary care data: a comparison with outcomes measured using the CIS-R.* BMJ Open, 2016. **6**(12).

3. Wang, Y., et al., *Do men consult less than women? An analysis of routinely collected UK general practice data.* BMJ Open, 2013. **3**(8).

4. Brilleman, S.L. and C. Salisbury, *Comparing measures of multimorbidity to predict outcomes in primary care: a cross sectional study.* Fam Pract, 2012.

5. Cornish, R.P., et al., *Socio-economic position and childhood multimorbidity: a study using linkage between the Avon Longitudinal study of parents and children and the general practice research database.* International Journal for Equity in Health, 2013. **12**(1): p. 66.

6. Atkinson, M.D., et al., *Development of an algorithm for determining smoking status and behaviour over the life course from UK electronic primary care records.* BMC Medical Informatics and Decision Making, 2017. **17**(1): p. 2.

7. Mukherjee, M., et al., *Estimating the incidence, prevalence and true cost of asthma in the UK: secondary analysis of national stand-alone and linked databases in England, Northern Ireland, Scotland and Wales—a study protocol.* BMJ Open, 2014. **4**(11).

8. Cornish, R.P., et al., *Using linked educational attainment data to reduce bias due to missing outcome data in estimates of the association between the duration of breastfeeding and IQ at 15 years.* International Journal of Epidemiology, 2015. **44**(3): p. 937-945.
